# Health‐promoting pedagogy: Using reflexivity to support learning and action in planetary health education

**DOI:** 10.1002/hpja.648

**Published:** 2022-09-06

**Authors:** Amy Christine Hickman, Rebecca Lauren Johnson, Sheleigh Patricia Lawler

**Affiliations:** ^1^ School of Public Health The University of Queensland Brisbane QLD Australia

**Keywords:** education, health promotion, planetary health, reflexivity, teaching

## Abstract

**Issue Addressed:**

International competencies for health promotion education require ethical practice that is supported by reflexive health promotion practitioners, yet professional bodies do not codify how health promotion curriculum should support students' skill development in reflexive practice.

**Methods:**

Reflexivity in teaching and learning was scaffolded through short, progressive reflective blogs assessments, supportive feedback/feedforward mechanisms, and nested assessment design.

**Results:**

Student feedback is offered to demonstrate the impact of reflexive pedagogy in health promotion education.

**Conclusion:**

Reflexivity in teaching and learning supports students in learning the role of health promotion in planetary health and developing skills in planetary health advocacy.

**So What:**

Explicitly teaching the practice and process of critical reflexivity can help students develop personal insight, support professional practice, and promote positive change in the health of people and our planet.

## 1 INTRODUCTION: CRITICAL REFLEXIVITY IN HEALTH PROMOTION EDUCATION

Modern health promotion positions human health as shaped by social and structural determinants and has evolved from a focus on individual health to addressing the health of communities,^1^, ^2^ strength‐based approaches^3^ and place‐based interventions.^4^ In the 21st century, health promotion has been challenged to reformulate itself to meet current challenges and the complexity of planetary health shaped by multiple and intersecting inequities.^5^ In order to address these challenges, health promoters must employ new ways of thinking about health, develop effective advocacy strategies and deliver innovative planetary health interventions to support both the health of human communities and the planet we depend upon for our survival.^5^, ^6^

As the contemporary health promotion practitioner undertakes this complex work, they must also respond to the IUHPE core competencies' call for ethical practice supported by reflexive health promotion practice (International Union for Health Promotion and Education, 2016).^7^ Reflexivity is defined as a tiered process of self‐reflection regarding past actions to gain insight to apply to future action.^8^ Critical reflexivity extends self‐reflection to ‘[challenge] the practices, roles, beliefs and values of the [health promotion] practitioner to promote learning and redevelopment of practice’^8^ ^(p539)^. Critical reflexivity is thus transformative, and can address systemic racism, social injustice, health inequities and the exploitation of the natural world.^9^, ^10^ As such, skills in critical reflexivity are needed to support transformative learning in health promotion and its application to supporting planetary health.^5^ Yet, despite the provision of competencies, professional bodies do not codify how health promotion curriculum should support student development in this endeavour.^11^

In response to this challenge, an ‘Introduction to Health Promotion’ course (PUBH7034) was redesigned as one of three courses in the health promotion stream within the Master of Public Health (MPH) program.

This brief report seeks to describe curricular choices and pedagogical strategies employed in the design and delivery of PUBH7034 that support student development of reflexivity within health promotion and planetary health. In particular, innovation in health promotion pedagogy is demonstrated through the strategic positioning of reflexivity to scaffold student learning and empower students to take risks to promote and advocate for planetary health. Methods are described through detailing the use of evidence‐based pedagogical strategies. The results demonstrate impact through student reflections offered through Student Evaluation of Course and Teacher (SECaT) surveys. This project received exemption from The Human Ethics Review Board (2022/HE001242).

## 2 METHODS

In advance of IUHPE's 23rd World Conference and the Rotorua Statement: WAIORA: Promoting Planetary Health and Sustainable Development for All, held in 2019, the course assessment structure was redesigned to include three new assessments: a written report on a planetary health topic of the students' choosing; an advocacy letter; and three short, formative reflexive blog assessments. The reflexive blog assessments were designed to enable students to learn the process and practice of critical reflection to address the cognitive and emotive complexity^12^ inherent in health promotion practice addressing planetary health. Advocacy skills were taught in alignment with IUPHE Core Competencies to ‘Advocate for Health’ (IUHPE 2016, p.11).

### 2.1 Scaffolding reflexivity through progressive short writing assessments

#### 2.1.1 Blog 1: personal standpoint

In order to be reflexive health promotion practitioners, critical reflexivity must be taught explicitly as it is not an intuitive practice.^13^ The standpoint assessment asks students to identify their social identities and values and how these shape their personal and professional choices. To scaffold this assessment, teaching staff modelled reflexivity through presenting their own written standpoint exemplars. This formative assessment was marked on a pass/nonpass basis, enabling students to practice reflexivity in a low‐stakes environment, developing their confidence and ability to take risks in developing their reflexivity skills.^14^

#### 2.1.2 Blog 2: reflexivity in practice

For the second reflexivity blog, a framework was introduced to scaffold students' ability to develop and communicate their reflective practice.^15^ The use of reflexivity frameworks enabled a diverse cohort to have a ‘common vocabulary and conceptual apparatus’ to engage in reflexivity and apply to their own learning styles^16^ ^(p500)^.

#### 2.1.3 Blog 3: reflexivity in research and action

Critical reflection is positioned as the first step to engaging action in health promotion.^17^ To prepare for the final advocacy letter assessment, students reflected in their third blog how the planetary health research undertaken as part of their written report impacted them professionally and personally. This reflection informed their call to action, and their approach to targeting an individual with power in that area.

### 2.2 Reflexivity through feedback processes

**FIGURE 1:**
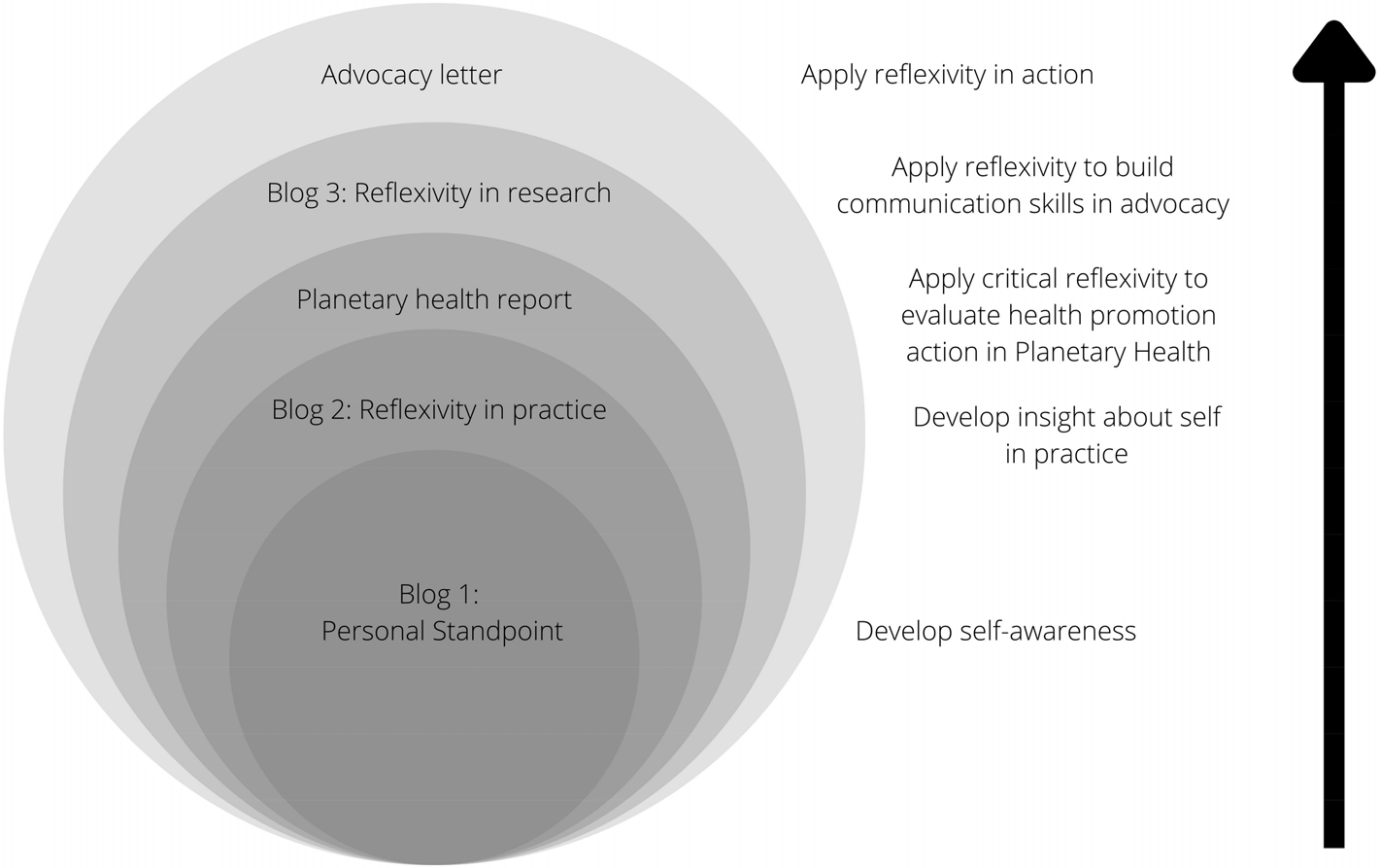
Nested assessment structure

A ‘nested’ or ‘multi‐stage’ structure of assessments is recognised as an effective device to promote student engagement with, and uptake of, feedback.^18^ The process enables students to develop skills in evaluative judgement as they engage in academic decision‐making and apply both internal (self‐reflective) and external (teaching staff) feedback to enhance their work.^19^ The nested assessment structure implemented in PUBH7034 (see Figure 1) allowed for the creation of valuable feedback loops for students. Weaving personal reflection together with external feedback given by teaching staff, students developed skills in evaluative judgement to support future tasks.^20^, ^21^ Nested assessments provided a foundation built through discursive reflexivity to support students' planetary health research and action.

### 2.3 Nested assessments support reflection on research leading to action

Students' first major task was a written report on a planetary health topic of their choosing. Eco‐social frameworks^22^ were presented to challenge the often fragmented and siloed approach to health promotion by making clear the inextricable linkages between human action, the health of planet and human health. Students' research goals were to evaluate current health promotion actions found in the literature at any level, in local, community or policy contexts.

After the written research report, students reflected *back* on their research and *forward* to plan for their advocacy letter, their final assessment (see description of Blog 3 above). Reflexivity enabled students to communicate in their advocacy letter why this issue was important to them, and to identify with the health promotion frameworks supporting action in the local or regional contexts.

## 3 RESULTS

Teaching strategies are evaluated in part by how students respond to them, thus student responses are offered as tangible results of our pedagogical methods. Students offered formal feedback on overall course design and curricula through the university's SECaT surveys. Feedback in relation to reflexive pedagogy and assessments is offered here.

### 3.1 Teaching reflexivity frameworks through scaffolded reflexive writing assessments

Students responded favourably to the reflexivity frameworks, ‘learning about the difference between light and dark reflexivity also helped me to improve my reflexivity skills’ (Student feedback, 2021).

*Not only are we equipped with the foundations to be confident, inquisitive and engaged health promotion practitioners, but also active citizens and advocates … [in a] supportive environment, where we were all encouraged to share our perspectives as we learned. (Student feedback, 2021)*

This feedback underscores how progressive reflective assessments can support students' understanding of their implicit biases, and develop self‐awareness needed to engage in ethical and culturally safe health promotion practice and research (IUHPE, 2016).

### 3.2 Nested assessment structure enabling feedback/feedforward processes

Students indicated the multi‐stage, progressive assessment design of the reflexivity blogs was an ‘…opportunity to receive feedback and be able to respond to comments in following assessment. The reflexivity tasks in particular were helpful for my learning’ (Student feedback, 2021).

In relation to the nested structure of the written report, reflexivity on research and action, and the advocacy letter, students described this design feature as supportive of their learning:

*I also liked that the assessments lead into one another. This added a difficult element for planning, as students needed to make choices that would work across more than one assessment, but this is a good skill to develop and added to the depth of learning on the chosen topic. (Student feedback, 2021)*

### 3.3 Advocacy informed by reflexivity

Advocacy skills were scaffolded through practicing and applying reflexivity to research and action. Students were encouraged to send their advocacy letters, and many felt empowered to do so. The impact of this agency is poignantly clear in one student's feedback:

*I feel as though studying health promotion actually enhanced my health, that the learning environment was supportive and encouraging so I was prepared to take some risks and explore challenging topics. This supportive environment meant I now have a better understanding of Australian Indigenous peoples' contributions to research and their belief systems. I also have a better connection to nature and feel more empowered to make changes. (Student feedback, 2021)*

This authentic assessment allowed students, to step into an agentic space^23^ to advocate for change to support planetary health. Reflexivity enabled students to locate their own professional values in advocacy, recognised as a necessary first step to reframe planetary health policy initiatives.^24^

## 4 CONCLUSION

Using reflexive pedagogy in health promotion becomes health promoting in and of itself, as students develop skills to reflect and act to promote a supportive environment for human and planetary health. Students appreciate that reflexivity is an important mode of inquiry that can extend learning to action in academic, professional and life contexts. Creating a supportive environment for reflexivity through a nested assessment structure and scaffolding enables students to explore, take risks and exercise agency in their learning while navigating complexity in assessment tasks.

A desktop scan of postgraduate health promotion courses in Australia undertaken in April 2022 suggests teaching planetary health through the lens of health promotion is innovative. PUBH7034 embeds critical reflexivity as a defined process that can be applied to personal insight, professional practice, and in promoting real change in the health of people and our planet. Student feedback suggests teaching critical reflexivity as both a process and practice supports students as they apply these skills as emerging public health researchers and practitioners. This approach contributes to the development of future‐oriented, reflexive, health promotion practitioners capable to address the complexities of planetary health and advocate for change.

## CONFLICT OF INTEREST

The authors declare no conflict of interest.
